# 2083: What factors explain failure to meet clinical recommendations for preschool children’s screen-time?

**DOI:** 10.1017/cts.2017.145

**Published:** 2018-05-10

**Authors:** Amanda E. Staiano, Andrew T. Allen, E. Kipling Webster, Corby K. Martin

## Abstract

OBJECTIVES/SPECIFIC AIMS: The American Academy of Pediatrics (AAP) recommends that preschool-aged children spend no more than 2 hours/day using digital screens such as TVs. However, there is a proliferation of digital screens in children’s daily lives both at school and at home. The purpose of this study was to examine factors that contribute to children’s screen-time, including their demographic characteristics and whether or not they have screen-time at school. METHODS/STUDY POPULATION: In total, 59 children (3.3±0.4 years of age; 47% female) enrolled in 3 child care centers participated. Center directors reported school screen-time; 1 center was classified as not providing screen-time and 2 centers were classified as providing screen-time. Parents reported child’s age, sex, and maternal education as a proxy for socio-economic status. Parents reported child’s out-of-school screen-time by responding to the question “During the past 30 days, on average how many hours per day did your child sit and watch TV or videos outside of school?” Additional questions queried how many hours per day did the child “use a computer or play computer games,” “play video games,” “use a smartphone,” and “use an iPad or tablet.” Children’s height and weight were collected using standard clinic procedures and body mass index (BMI) was calculated. *T* tests were used to examine differences in screen-time by age, sex, and school screen-time. General linear models were used to examine the influence of school screen-time (1=no screen-time, 0=between 1 and 60 min/day of screen-time), age, BMI, and maternal education on out-of-school screen-time and time spent with each device. Logistic regression analysis was used to examine likelihood of meeting screen-time recommendations based on the same characteristics. RESULTS/ANTICIPATED RESULTS: Parent-reported total screen-time was 6.3±3.6 hours/day (h/d); specifically, 2.5±1.1 h/d watching TV, 1.5±2.2 h/d using a smartphone, 1.1±0.9 h/d using a tablet, 0.8±1.0 h/d on a computer, and 0.5±0.7 h/d playing video games. Based on total screen-time, 15% of children met AAP recommendations; based on TV viewing only, 52% met AAP recommendations. The 4-year-old children viewed more screen-time overall compared to the 3-year-old children including on TV, computer, and tablet (*p*<0.05), but there were no sex differences. In fully adjusted linear models, out-of-school screen-time was lower among those who had no screen-time at school (*p*=0.02) and higher among older children (*p*<0.01). Computer use was higher among older children (*p*=0.02). Older children and those with lower maternal education were less likely to meet clinical recommendations based on TV viewing (*p*<0.05). There were no observed associations with likelihood of meeting clinical recommendations based on total screen-time. BMI was not a significant predictor of screen-time. DISCUSSION/SIGNIFICANCE OF IMPACT: The majority of children exceeded AAP screen-time limits, with screen-time sharply higher among older children, and the associations did not vary by weight status. Children who attended schools that allowed screen-time had higher amounts of out-of-school screen-time. Pediatricians and healthcare providers should query parents on children’s screen-time practices at home and at school and offer strategies to help families meet the clinical recommendations.

